# Age- and Hypertension-Related Changes in NOS/NO/sGC-Derived Vasoactive Control of Rat Thoracic Aortae

**DOI:** 10.1155/2022/7742509

**Published:** 2022-03-09

**Authors:** Andrea Berenyiova, Peter Balis, Michal Kluknavsky, Iveta Bernatova, Sona Cacanyiova, Angelika Puzserova

**Affiliations:** Institute of Normal and Pathological Physiology, Centre of Experimental Medicine Slovak Academy of Sciences, Bratislava 84104, Slovakia

## Abstract

This study was aimed at examining the role of the NOS/NO/sGC signaling pathway in the vasoactive control of the thoracic aorta (TA) from the early to late ontogenetic stages (7 weeks, 20 weeks, and 52 weeks old) of normotensive Wistar-Kyoto (WKY) rats and spontaneously hypertensive rats (SHRs). Systolic blood pressure (SBP) and heart rate (HR) were significantly increased in SHRs compared to age-matched WKYs, which was associated with left heart ventricle hypertrophy in all age groups of rats. The plasma urea level was increased in 20-week-old and 52-week-old SHRs compared with WKYs without increasing creatinine and uric acid. The total cholesterol levels were lower in 20-week-old and 52-week-old SHRs than in WKYs, but triglycerides were higher in 7-week-old SHRs. The fructosamine level was increased in 52-week-old SHRs compared with age-matched WKYs and unchanged in other age groups. Superoxide production was increased only in 7-week-old SHRs compared to age-matched WKYs. The endothelium-dependent relaxation (EDR) of the TA deteriorated in both rat strains during aging; however, endothelial dysfunction already occurred in 20-week-old SHRs and was even more enhanced in 52-week-old rats. Our results also demonstrated increased activity of NOS in 52-week-old WKYs. Moreover, 7-week-old and 52-week-old WKY rats displayed an enhanced residual EDR after L-NMMA (NOS inhibitor) incubation compared with 20-week-old rats. Our results showed that in 7-week-old SHRs, the residual EDR after L-NMMA incubation was increased compared to that in other age groups. The activity of NOS in the TA was comparable in 7-week-old and 20-week-old SHRs, but it was reduced in 52-week-old SHRs compared to younger SHRs and 52-week-old WKYs. Thus, it seems that, in contrast to SHRs, the NOS/NO system in WKYs is probably able to respond to age-related pathologies to maintain endothelial functions and thus optimal BP levels even in later periods of life.

## 1. Introduction

Aging and age-related processes have become important issues in recent cardiovascular studies since they significantly deteriorate the quality of life of older populations [1]. There is an obvious correlation between cardiovascular outcomes and aging. Several structural, functional, and molecular changes in the cardiovascular system have been reported in aged individuals. The process of aging involves, among others, an increased production of free radicals and fluid shear stress, endothelial dysfunction, and vascular remodeling. These pathological mechanisms lead to enhanced vasoconstriction, reduced vasorelaxation, vascular hypertrophy, and rigidity of the vessel wall [2].

Alterations similar to aging were confirmed under conditions of elevated arterial blood pressure (BP)—hypertension. Previous studies using an animal model of arterial hypertension, spontaneously hypertensive rats (SHRs), demonstrated an increased contractile response after stimulation of adrenergic receptors in both conduit and resistant arteries [3] mainly as a consequence of hyperactivity of the sympathetic nervous system [4]. Moreover, morphological studies revealed that along with BP elevation, the arterial wall area increased and was hypertrophied in all parts of the arterial tree, in the thoracic aorta (TA), carotid artery, and iliac artery in adult SHRs [5].

Endothelial dysfunction is a common hallmark of age- and hypertension-related pathologies. This is a term that covers the imbalance between the vasoconstrictor and vasorelaxant agents produced by endothelial cells [6]; moreover, it is associated with reduction of their anticoagulant and antithrombotic properties, acceleration of vascular growth, and remodeling [7]. Nitric oxide (NO) is a crucial molecule of the arterial wall with a significant vasorelaxant effect. It has also been shown that NO represents the dominant mediator of endothelium-dependent vasorelaxation in TA [8]. Endothelial NO rapidly diffuses to vascular smooth muscle cells (VSMCs) and initiates the production of cyclic guanosine monophosphate (cGMP) due to the activity of soluble guanylate cyclase (sGC). Recently, sGC has proven to be a key enzyme in the treatment of various cardiovascular pathologies since sGC stimulators and activators are in preclinical and clinical development for the treatment of pulmonary hypertension, which has been reported as a new and promising milestone in the field of NO/sGC/cGMP pharmacology [9].

Our previously performed studies related to the participation of endogenous NO in the vasoregulatory mechanism in the early stage of SHR ontogenesis revealed that the NO-dependent vasorelaxant component of the TA in 4-week-old SHRs was enhanced compared with that in age-matched normotensive controls. We also speculated that TA could be endowed with a unique predisposition to decreased contractility and strengthened endothelium-regulated vasorelaxant mechanisms that serve as adaptive mechanisms during the fully developed phase of hypertension [10]. On the other hand, in 7-week-old SHRs, we showed reduced endothelium-dependent relaxation associated with a reduction in NO-independent mechanisms in femoral arteries accompanied by increased systolic BP. However, the contribution of NO to vasorelaxation was significant and comparable to that in normotensive rats [11]. In young adult SHRs (18-week-old), we found reduced maximal endothelium-dependent vasorelaxation and NO synthase (NOS) activity in the aorta compared with 4-week-old SHRs [12]. Similar findings of NOS activity were seen in 22-week-old SHR aortae compared with 7-week-old SHRs [10].

Based on this, it seems that the endogenous NOS/NO/sGC system and its involvement in the regulation of arterial tone are highly influenced by age. Therefore, the main aim of this study was to investigate the importance of NO and its participation in the vasomotor control of TA during aging and BP increase. The paper may offer a complex overview of the position of NO in endothelium-derived vasorelaxation and pathophysiology of essential hypertension focusing on three different age groups: young juvenile (7-week-old), young adult (20-week-old), and old (52-week-old) normotensive Wistar-Kyoto (WKY) rats and SHRs. In addition, we evaluated the levels of selected plasma parameters that could affect the function of vessels and superoxide production in TA as an important scavenger of vascular NO. We hypothesized that the reduced NO synthesis and/or bioavailability with altered oxidative status would produce reduced endothelium-dependent vasorelaxation and BP increases in the course of aging.

## 2. Materials and Methods

### 2.1. Guide for the Use and Care of Laboratory Animals

Procedures were performed in accordance with institutional guidelines and were approved by the State Veterinary and Food Administration of the Slovak Republic (decision no. Ro-3095/14-221 and decision no. Ro-1087/17-221) and by the Ethics Committee (project code EK/vekhyp/2014, approved 24 June 2014, and EK/1/17, approved 6 February 2017) according to the European Convention for the Protection of Vertebrate Animals Used for Experimental and Other Scientific Purposes, Directive 2010/63/EU of the European Parliament. All rats used in the study were born in an accredited breeding establishment of the Institute of Normal and Pathological Physiology, Centre of Experimental Medicine Slovak Academy of Sciences (INPP CEM SAS), and were housed in groups of 2 to 4 animals, each strain separately, under a 12 h light-12 h dark cycle (06:00–18:00 light phase), at a constant humidity (45-60%) and temperature (22-24°C), with free access to standard laboratory rat chow and drinking water. INPP CEM SAS provided veterinary care. 7-week-old (young juvenile rats), 20-week-old (young adult rats), and 52-week-old (old rats) males of normotensive Wistar-Kyoto (WKY, sublines WKY/NHsd, HARLAN UK) and spontaneously hypertensive rats (SHR, sublines SHR/NHsd, HARLAN UK) were included in the present study. Every phenotype was divided into three groups: 7-week-old (WKY-7, *n* = 10; SHR-7, *n* = 11), 20-week-old (WKY-20, *n* = 9; SHR-20, *n* = 10), and 52-week-old (WKY-52, *n* = 9; SHR-52, *n* = 14). The rats were killed by decapitation after brief CO_2_ anesthesia.

### 2.2. General Biometric and Cardiovascular Parameters

The systolic blood pressure (SBP) and heart rate (HR) were measured in prewarmed rats by noninvasive plethysmography on rat tail arteries before the beginning of the in vitro study (except handling); the body weight (BW) of the animal was also measured [13]. The weight of the left heart ventricle (free wall) (LV) and the length of the tibia (TL) were measured to evaluate organ hypertrophy [14, 15].

### 2.3. Basic Plasma Parameters

Trunk blood was collected just after decapitation in heparinized test tubes, and blood was immediately centrifuged (850 g, 10 min, 4°C). Plasma samples were frozen in aliquots for biochemical determinations and stored at -80°C until analysis. The basic levels of urea, creatinine, uric acid, total cholesterol, triglycerides (TAG), and fructosamines were commercially determined in the accredited veterinary laboratory Laboklin GMBH (Bratislava, Slovakia) using standard laboratory methods.

### 2.4. Measurement of Superoxide Production in Aortic Tissue

The assay was performed as described previously [16] with some modifications. The aortic rings were cut (10–15 mg), cleaned of connective tissue, and placed into cold modified physiological salt solution (PSS, in mmol/L: NaCl 118.99, KCl 4.69, NaHCO_3_ 25, MgSO_4_·7H_2_O 1.17, KH_2_PO_4_ 1.18, CaCl_2_·2H_2_O 2.5, Na_2_EDTA 0.03, and glucose 5.5, pH 7.4). Lucigenin (50 *μ*mol/L), as well as tissue samples alone, was added to PSS bubbled with pneumoxide (5% CO_2_ and 95% O_2_) at pH 7.4 and 37°C and preincubated in the dark for 20 min. After preincubation, either background chemiluminescence or chemiluminescence produced by the aortic rings was measured for 6 min using a TriCarb 2910TR liquid scintillation analyzer (PerkinElmer, Waltham, USA). Background counts were subtracted from values obtained from the samples. The results are expressed as counts per minute per mg of tissue (cpm/mg).

### 2.5. Functional Study

The isolated TA was cleaned of connective tissue using a microscope and cut into 5 mm length rings. The rings were vertically fixed between 2 stainless wire triangles and immersed in a 20 mL incubation organ bath with PSS. This solution was oxygenated with a mixture of 95% O_2_ and 5% CO_2_ and kept at 37°C. The upper triangles were connected to sensors of isometric tension (FSG-01, MDE, Budapest, Hungary), the changes in tension were registered by an AD converter NI USB-6221 (National Instruments, Austin, USA, MDE, Budapest, Hungary), and the changes in isometric tension were registered by using DEWEsoft (Dewetron, Prague, Czech Republic) and SPEL Advanced Kymograph (MDE, Budapest, Hungary) software. A resting tension of 1 g was applied to each ring and maintained throughout a 45- to 60 min equilibration period until stress relaxation no longer occurred.

The relaxant responses were followed on rings precontracted with phenylephrine (Phe; 10^−6^ mol/L) after achieving a stable plateau of contraction. The isolated TA rings were then exposed to cumulative concentrations of acetylcholine (ACh; 10^−9^ − 3×10^−5^ mol/L). The rate of relaxation was expressed as a percentage of the Phe-induced contraction.

The participation of the endogenous NO system in the vasomotor responses of TA was followed before and 30 minutes after pretreatment with a nonspecific inhibitor of NOS, N^G^-methyl-L-arginine acetate salt (L-NMMA; 10^−4^ mol/L), to block basal and receptor-induced endogenous NO production. To confirm the involvement of the soluble guanylate cyclase (sGC) signaling pathway in ACh-induced relaxation, a sGC inhibitor, 1H-[1,2,4]oxadiazole[4,3-a]quinoxalin-1-one (ODQ, 10^−5^ mol/L), was applied 30 min before the addition of the contractile agonist.

All drugs were dissolved in distilled water, except ODQ, which was dissolved in dimethylsulfoxide. All concentrations are expressed as the final concentrations in the organ chamber.

The effect of the age and hypertension on the endothelium-dependent relaxation (EDR) is expressed separately using the same concentration-dependent ACh curves from all animals in each group, before and after application of inhibitors, respectively.

### 2.6. Nitric Oxide Synthase Activity

The aortae were carefully dissected, and connective tissue was removed. NOS activity was determined in 20% fresh tissue homogenates (*w* : *v*) as described in detail previously [17]. Briefly, the aortic tissue was immersed in ice-cold buffer (0.05 mol/L Tris-HCl, pH 7.4) containing 1% protease inhibitor cocktail. After homogenization at 4°C (Ultra-Turrax homogenizer) and centrifugation of homogenates (4°C, 10 min, 3000 g), NOS activity was determined in the supernatants by the conversion of [3H]-L-arginine (specific activity 5 GBq/mmoL, ~100,000 dpm, ARC, St. Louis, USA) to [3H]-L-citrulline in the presence of 50 mmol/L Tris/HCl (pH 7.4), containing NOS cofactors in a total volume of 100 *μ*L. The reaction was stopped after 20 min of incubation by adding 1 mL of ice-cold stop solution containing 20 mmoL 4-(2-hydroxyethyl)-1-piperazineethanesulfonic acid (pH 5.5) and NOS inhibitors. One milliliter of the final mixture was applied to Dowex 50 WX-8 columns (Na^+^-form), and [3H]-L-citrulline was eluted by adding deionized water. The results were determined by liquid scintillation counting (TriCarb 2910TR, PerkinElmer, Waltham, USA). NOS activity was expressed as pkat per gram of protein. The protein concentration was determined using the Lowry method [18].

### 2.7. Statistical Analysis

The data were expressed as means ± SEM. For the statistical evaluation of differences between groups, a two- and three-way analysis of variance (ANOVA) with the Bonferroni post hoc test was used. The differences between means were considered significant at *p* < 0.05. OriginPro2019b (OriginLab, Northampton, USA), GraphPad Prism 5.0 (GraphPad Software; San Diego, USA), and Statistica 13.5 (StatSoft; Hamburg, Germany) were used for the statistical analyses.

### 2.8. Drugs

All drugs were purchased from Sigma-Aldrich (Bratislava, Slovakia) unless otherwise stated.

## 3. Results

### 3.1. General Biometric and Cardiovascular Parameters

Table 1 summarizes the differences in SBP and other basic biometric and cardiovascular parameters among the experimental groups. The BW of the animals increased with age in both WKYs and SHRs; however, the 52-week-old SHRs had a significantly lower BW than the age-matched WKYs. On the other hand, SHRs revealed an elevated SBP compared to WKYs in all ontogenetic stages. In SHRs, the SBP was comparable between the young adult and old rats, but it was significantly higher compared to young juvenile 7-week-old animals. There was also an age-dependent increase in SBP in WKY rats; in 52-week-old rats, we observed significantly higher SBP than the other age groups of this strain. The HR of SHRs was higher than that of WKYs in all age groups, but there were no changes in this parameter during ontogenesis in either SHRs or WKYs. By age, the weight of LV was increased in both strains; moreover, in SHRs, the weight of LV was higher than that in age-matched WKY rats, except in the 7-week groups. An age-dependent increase was also observed in TL in both WKYs and SHRs. The length of the tibia was decreased in 20-week-old SHRs compared to WKYs of the same age; in 52-week-old rats, there were no differences between the strains in TL. Generally, in SHRs, the LV/BW ratio and LV/TL were increased compared to those in WKYs.

### 3.2. Selected Plasma Parameters

**Figure 1:**
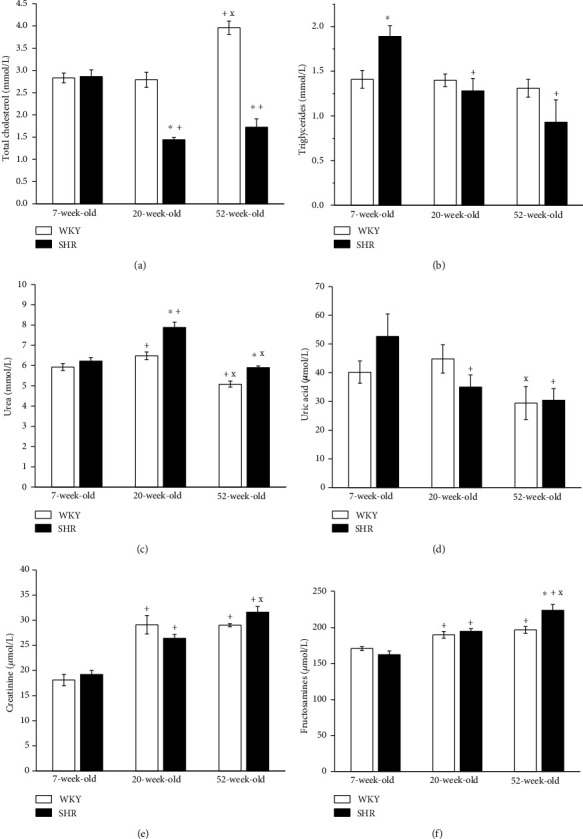
The effect of the age and phenotype on selected plasma parameters. WKY: Wistar-Kyoto rats; SHR: spontaneously hypertensive rats. The results are presented as the mean ± SEM of *n* = 5–10 rats, and differences between groups were analyzed by two-way analysis of variance (ANOVA) with the Bonferroni post hoc test on ranks. ^∗^*p* < 0.05 vs. WKY at the same age (WKY-7 vs. SHR-7, WKY-20 vs. SHR-20, and WKY-52 vs. SHR-52), ^+^*p* < 0.05 vs. 7-week group of the same phenotype (WKY-20 vs. WKY-7, SHR-20 vs. SHR-7, WKY-52 vs. WKY-7, and SHR-52 vs. SHR-7), ^x^*p* < 0.05 vs. 20-week group of the same phenotype (WKY-52 vs. WKY-20 and SHR-52 vs. SHR-20).

In WKY rats, the highest urea level was measured in 20-week-old rats, which decreased with age. The plasma levels of creatinine were similarly increased from the 20^th^ week of age, and there was a significant reduction in uric acid in 52-week-old WKY rats compared with 20-week-old rats. The total cholesterol in plasma was increased only in 52-week-old WKY rats, and the levels of fructosamines were also increased in young adult and old normotensive rats compared to young juvenile rats. In SHRs, the plasma level of urea was increased in 20-week-old rats compared to young juveniles, and it was reduced in 52-week-old rats compared with young adults but not with young juvenile rats. Creatinine in plasma increased with age, and uric acid was reduced in 20-week-old and 52-week-old SHRs. The results demonstrated significantly reduced plasma levels of total cholesterol and TAG in young adult and old SHRs, and the levels of fructosamines increased with the age of the animals (Figure 1).

Our results revealed a significantly increased urea level in 20-week-old and 52-week-old SHRs compared with age-matched WKYs; on the other hand, there were no differences between the strains in terms of plasma levels of creatinine and uric acid (Figure 1). Moreover, the levels of total cholesterol were significantly lower in 20-week-old and 52-week-old SHRs than in WKYs; however, we recorded an increased TAG level in 7-week-old SHRs. The fructosamine level was increased in 52-week-old SHRs compared with age-matched WKYs and unchanged in other age groups.

### 3.3. Superoxide Production in the Aortic Tissue

**Figure 2:**
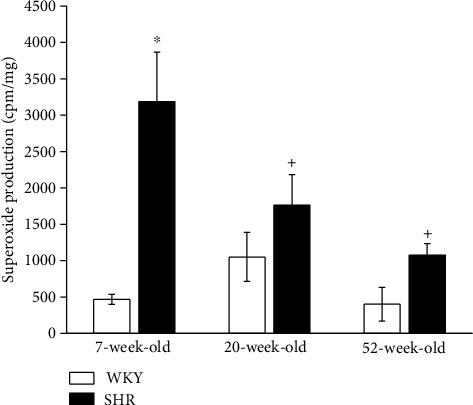
The effect of the age and phenotype on the production of superoxide in the aorta. WKY: Wistar-Kyoto rats; SHR: spontaneously hypertensive rats. The results are presented as the mean ± SEM of *n* = 5–9 rats, and differences between groups were analyzed by two-way analysis of variance (ANOVA) with the Bonferroni post hoc test on ranks. ^∗^*p* < 0.05 vs. WKY at the same age (WKY-7 vs. SHR-7), ^+^*p* < 0.05 vs. 7-week group of the same phenotype (SHR-20 vs. SHR-7 and SHR-52 vs. SHR-7).

While the production of superoxide in WKY rats was unchanged by age, in SHRs, the superoxide levels were decreased in 20- and 52-week-old rats. Superoxide production was increased in 7-week-old SHRs compared to age-matched WKYs, and there was no significant difference in the other age groups (Figure 2).

### 3.4. Vasoactive Responses of Isolated Aortic Rings

#### 3.4.1. Age-Related Differences in Endothelium-Dependent Vasorelaxation in WKYs and SHRs

**Figure 3:**
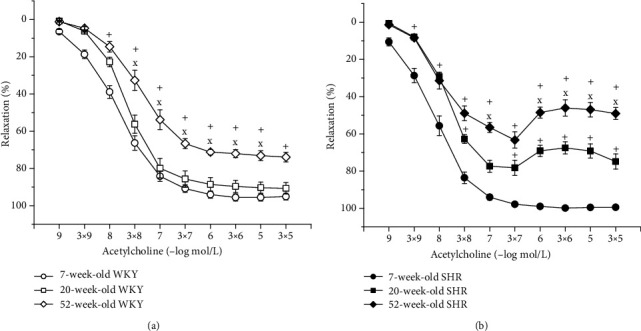
The effect of the age on the endothelium-dependent vasorelaxation of the thoracic aorta in Wistar-Kyoto (WKY) (a) and spontaneously hypertensive rats (SHR) (b). The results are presented as the mean ± SEM of *n* = 8–10 rats, and differences between groups were analyzed by two-way analysis of variance (ANOVA) with the Bonferroni post hoc test on ranks. ^+^*p* < 0.05 vs. 7-week-old rats, ^x^*p* < 0.05 vs. 20-week-old rats.

The EDR amplitude was significantly age-dependent in both strains (WKY: *F*_(2,268)_ = 149.77, *p* < 0.0001, SHR: *F*_(2,288)_ = 449.56, *p* < 0.0001). The EDR was comparable between the 7-week and 20-week WKYs. In 52-week-old WKYs, the EDR was significantly reduced compared to the other age groups (Figure 3(a)). On the other hand, reduced vasorelaxation of the TA in SHRs was observed in 20-week-old rats (compared to 7-week-old SHRs), which was more pronounced in 52-week-old SHRs than in both 7-week-old and 20-week-old SHRs (Figure 3(b)).

#### 3.4.2. Hypertension-Related Differences in Endothelium-Dependent Vasorelaxation between Age-Matched WKYs and SHRs

**Figure 4:**
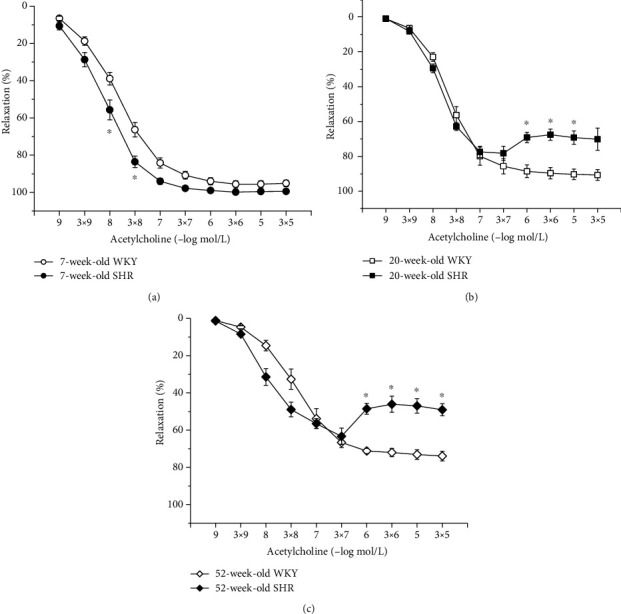
The effect of hypertension on the endothelium-dependent vasorelaxation of the thoracic aorta in 7-week-old (a), 20-week-old (b), and 52-week-old (c) rats. WKY: Wistar-Kyoto rats; SHR: spontaneously hypertensive rats. The results are presented as the mean ± SEM of *n* = 8–10 rats, and differences between groups were analyzed by two-way analysis of variance (ANOVA) with the Bonferroni post hoc test on ranks. ^∗^*p* < 0.05 vs. WKY.

In 7-week-old SHRs, the EDR was significantly enhanced (*F*_(1,218)_ = 52.37, *p* < 0.0001; Figure 4(a)) compared with that in the WKY. However, a deterioration of the vasorelaxant ability of the TA in SHRs already appeared in 20-week-old rats (*F*_(1,138)_ = 23.69, *p* < 0.0001; Figure 4(b)), and we also observed significant endothelial dysfunction in 52-week-old SHRs (*F*_(1,198)_ = 18.95, *p* < 0.0001; Figure 4(c)).

#### 3.4.3. Age-Related Changes in NO Synthase and Soluble Guanylate Cyclase Participation in Endothelium-Dependent Vasorelaxation in WKYs and SHRs

**Figure 5:**
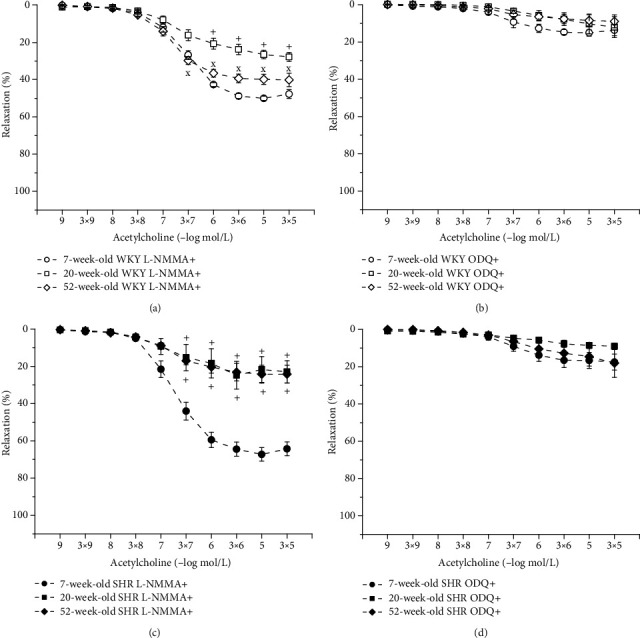
The effect of the age on participation of NO synthase (a, c) and soluble guanylate cyclase (b, d) in the endothelium-dependent vasorelaxation of the thoracic aorta in Wistar-Kyoto (WKY) and spontaneously hypertensive rats (SHR). The results are presented as the mean ± SEM of *n* = 8–10 rats, and differences between groups were analyzed by two-way analysis of variance (ANOVA) with the Bonferroni post hoc test on ranks. ^+^*p* < 0.05 vs. 7-week-old, ^x^*p* < 0.05 vs. 20-week-old rats.

To test the participation of the NO/NOS and sGC systems in EDR, we incubated the aortic rings with L-NMMA and ODQ. In normotensive rats, acute NOS inhibition reduced the EDR to the greatest extent in 20-week-old rats compared to 7-week-old and 52-week-old rats; however, there were no differences in the EDR after L-NMMA incubation between young (7-week-old) and old (52-week-old) rats (*F*_(2,138)_ = 77.69, *p* < 0.0001; Figure 5(a)). The inhibition of sGC significantly blocked the EDR, similarly in every age group (Figure 5(b)). Our results demonstrated that the participation of the NO/NOS system in the EDR was affected by the age of SHRs. While in young adults and old SHRs, L-NMMA incubation significantly inhibited the EDR, in young juvenile rats, we observed a significant residual EDR despite NOS inhibition (*F*_(2,148)_ = 114.62, *p* < 0.0001; Figure 5(c)). Incubation with ODQ significantly inhibited the EDR regardless of age (Figure 5(d)).

#### 3.4.4. Hypertension-Related Differences in NO Synthase and Soluble Guanylate Cyclase Participation in Endothelium-Dependent Vasorelaxation between Age-Matched WKYs and SHRs

**Figure 6:**
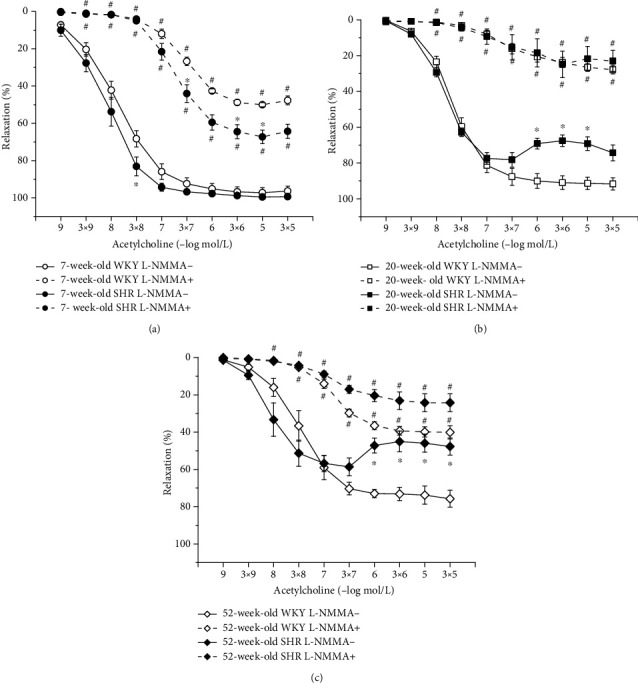
The effect of hypertension on NO synthase participation in endothelium-dependent vasorelaxation of the thoracic aorta in 7-week-old (a), 20-week-old (b), and 52-week-old (c) rats. WKY: Wistar-Kyoto rats; SHR: spontaneously hypertensive rats. The results are presented as the mean ± SEM of *n* = 8–10 rats, and differences between groups were analyzed by three-way analysis of variance (ANOVA) with the Bonferroni post hoc test on ranks. ^∗^*p* < 0.05 vs. WKY, ^#^*p* < 0.05 vs. EDR without L-NMMA.

**Figure 7:**
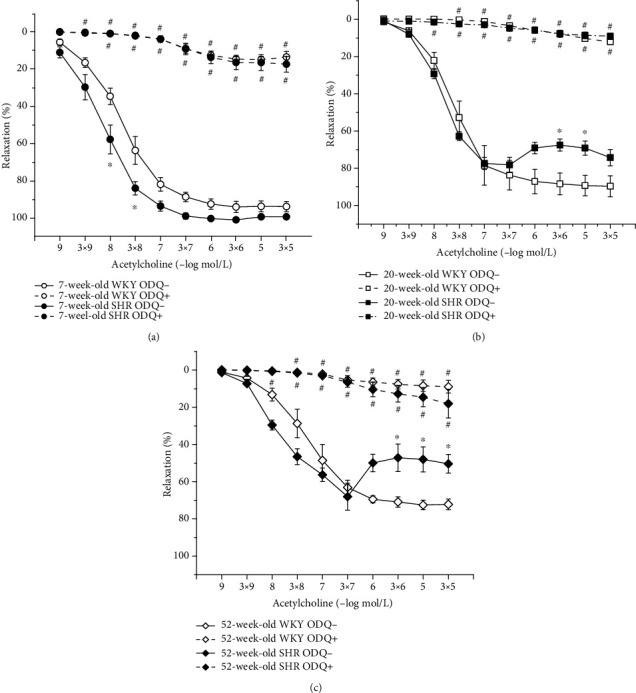
The effect of hypertension on sGC participation in endothelium-dependent vasorelaxation of the thoracic aorta in 7-week-old (a), 20-week-old (b), and 52-week-old (c) rats. WKY: Wistar-Kyoto rats; SHR: spontaneously hypertensive rats. The results are presented as the mean ± SEM of *n* = 8–10 rats, and differences between groups were analyzed by three-way analysis of variance (ANOVA) with the Bonferroni post hoc test on ranks. ^∗^*p* < 0.05 vs. WKY, ^#^*p* < 0.05 vs. EDR without ODQ.

We observed a relatively well-preserved EDR in 7-week-old rats after L-NMMA incubation in both strains; however, in SHRs, this residual relaxation was significantly larger than in age-matched WKYs (phenotype: *F*_(1,228)_ = 57.28, *p* = 0.0001; inhibition: *F*_(1,228)_ = 1934.32, *p* < 0.0001; concentration × phenotype × inhibition: *F*_(9,228)_ = 3.26, *p* = 0.001; Figure 6(a)). Although endothelial dysfunction appeared in 20-week-old rats, the effect of acute NOS inhibition on the EDR was comparable between the two phenotypes (Figure 6(b)). In 52-week-old rats, there was a significant effect of the phenotype × inhibition interaction on the EDR (*F*_(1,118)_ = 6.79, *p* = 0.01); in old SHRs, the residual EDR after acute L-NMMA application was smaller than that in WKYs (Figure 6(c)). Our results demonstrated that sGC is significantly involved in the EDR regardless of the phenotype and age (Figures 7(a)–7(c)).

#### 3.4.5. Nitric Oxide Synthase Activity

**Figure 8:**
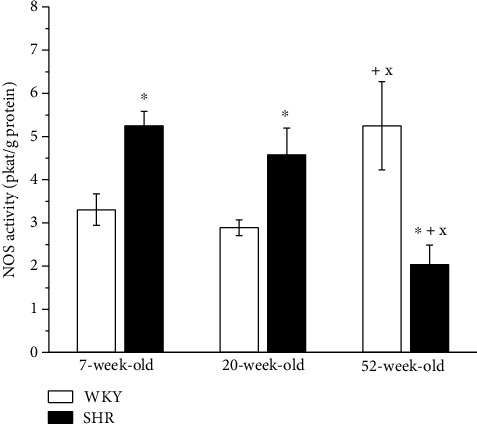
The effect of the age and phenotype on the NO synthase activity in the aorta. WKY: Wistar-Kyoto rats; SHR: spontaneously hypertensive rats. The results are presented as the mean ± SEM of *n* = 6 rats, and differences between groups were analyzed by two-way analysis of variance (ANOVA) with the Bonferroni post hoc test on ranks. ^∗^*p* < 0.05 vs. WKY at the same age, ^+^*p* < 0.05 vs. 7-week-old of the same phenotype, ^x^*p* < 0.05 vs. 20-week-old rats of the same phenotype.

The activity of NOS in the aorta was comparable between the 7-week-old and 20-week-old WKY rats, but it was significantly increased in 52-week-old normotensive rats. On the other hand, in SHRs, NOS activity was decreased in 52-week-old rats compared with the other age groups. In 7-week-old and 20-week-old SHRs, NOS activity was significantly increased compared with that in WKYs; however, in 52-week-old rats, reduced NOS activity was observed (Figure 8).

## 4. Discussion

The present study provides an overview of the vasoactive manifestation of the NOS/NO/sGC signaling pathway in the TA from the early to late ontogenetic stages of normotensive WKYs and SHRs. Moreover, the effect of aging and increased blood pressure on selected plasma parameters and superoxide production in these rat strains was investigated. The main limitation of the present study is the lack of the morphological confirmation of the physiological/biochemical analyses. There is clear evidence that the structural remodeling of the arterial wall significantly modifies the vasoactive properties of the vessels, including their vasorelaxant abilities [12], which has to be kept in mind.

SHRs are a widely used experimental model of human arterial hypertension. According to the literature, BP in SHRs starts to increase from 6 weeks of age, and the continual increase in SBP stops at approximately 36 weeks of age [19]. Our results are in agreement with these data, since we observed a significantly increased SBP in all investigated age groups of SHRs compared with age-matched WKYs; nevertheless, the SBP values were comparable in 20- and 52-week-old SHRs. The increased SBP in SHRs was also associated with increased HR and left heart ventricle hypertrophy in all age groups investigated. The main morphological alterations in SHRs, such as changes in the heart and arterial wall trophicity, start from approximately the 5^th^ week of life. Our previous morphological analysis revealed hypotrophy of the TA wall in 4-week-old SHRs compared to Wistar rats. On the other hand, in adulthood, together with a rapid BP increase, the TA was hypertrophied compared to normotensive rats [12].

In SHRs, the total cholesterol and TAG levels decreased with age; moreover, in 20- and 52-week-old SHRs, the level of total cholesterol was significantly lower than that in age-matched WKYs. In SHRs, an enhanced lipolysis has been suggested as a result of increased sympathetic outflow; moreover, an increased secretion of cholesterol combined with deficiencies in enteric capture and molecular transport of cholesterol was also demonstrated [20, 21]. In addition, it has been shown that the levels of cholesterol-related steroid hormones, such as corticosterone, were elevated already in 7-week-old female SHRs compared to age-matched WKYs, and in 10-week-old SHRs, the serum levels of progesterone, corticosterone, and cortisol were significantly elevated compared to those in 5-week-old SHRs and 10-week-old WKYs [16, 22], which may also contribute to the reduction in total cholesterol in our experimental group.

Indeed, it is known that a high plasmatic uric acid level is associated with an increased risk of cardiovascular diseases (CVDs), and endothelial dysfunction has been suggested as a potential mechanism involved in hyperuricemia-induced CVDs. In this study, we did not find increased uric acid levels in SHRs compared to age-matched WKYs. In our previous study, acute exposure of resistant mesenteric arteries, femoral arteries, and aortae isolated from aged WKY rats to a high concentration of uric acid did not provoke changes in endothelial function in these arteries [23]. We observed a decreasing uric acid concentration and EDR with age. Thus, the role of hyperuricemia in endothelial dysfunction in aged rats is not supported by our data.

Fructosamine concentration is often used as a marker of carbonyl stress. In our recent study, we reported that the plasma concentration of advanced glycation end-products (AGEs) was stably decreased in 20- and 52-week-old WKYs compared to 7-week-old WKYs; however, in SHRs, it was decreased only in 20-week-old SHRs, and there was no difference between the strains. We also reported that fructosamine concentrations were not significantly different between these individual age groups when expressed in molar concentration to gram of proteins [24]. On the other hand, we found a decreased superoxide production with age in SHRs, while in WKYs, superoxide production was comparable between the age groups. We surprisingly recorded significantly enhanced superoxide production only in young juvenile SHRs. Gomes et al. [25] reported increased H_2_O_2_ production in the renal cortex, although no differences were detected in urinary lipid peroxidation in 12-week-old SHRs. This finding was associated with an upregulation of NADPH oxidase and downregulation of antioxidant superoxide dismutase (SOD) 1 and SOD3 enzymes. However, 48-week-old WKYs and SHRs displayed comparable oxidant and antioxidant profiles. The authors suggested that this conflict with the current view that hypertension is a state of oxidative stress might arise from the fact that normotensive WKY developed obesity with aging, which could operate as a confounding factor of oxidative stress. In agreement, in our study, 52-week-old SHRs had a significantly lower BW (and comparable TL) than age-matched WKYs.

In the other part of our experiment, we examined the impact of age and hypertension on the EDR of TA. We incubated the aortic rings with a nonselective NOS inhibitor (L-NMMA) to assess the participation of NO in EDR during aging. The degree of EDR inhibition after L-NMMA is in agreement with the degree of NO participation in EDR; in general, the more enhanced inhibition is, the more significant NO participation. The vasorelaxant abilities of the TA deteriorated in both rat strains during aging; however, endothelial dysfunction already occurred in 20-week-old SHRs and was even more enhanced in 52-week-old rats. Similar results were reported by Matz et al. [26] in Wistar rats, who demonstrated an unchanged EDR between 12- to 14-old and 32-week-old normotensive rats, but a significant reduction was reported at 70-100 weeks of age. They also found increased endothelial NOS protein expression and a significant involvement of endothelial NO in the EDR, since N^G^-nitro-L-arginine (L-NA) abolished the response to ACh in the aorta. Our results also demonstrated a significantly increased total activity of NOS in 52-week-old WKYs compared to the other age groups. The increased NOS activity and the unchanged superoxide production during the aging indicated a high NO bioavailability in old WKY which was not, however, manifested in the vasoactive responses of TA, since we observed a reduced EDR in these rats. We assume that this fact could be associated with the prevalence of other negative impacts such as enhanced production of contractile agents, advanced structural remodeling that could eliminate the vasoactive manifestation of the preserved NO sources. Moreover, we showed that 7-week-old and 52-week-old WKY rats displayed an enhanced residual EDR after L-NMMA incubation compared with 20-week-old rats. Our results suggested that the greatest L-NMMA-sensitive component of EDR was present in 20-week-old WKY rats and the smallest in 52-week-old WKY rats. Incomplete inhibition of EDR after L-NMMA could be caused by a concentration of L-NMMA that only partly inhibited NOS and therefore was not maximally effective in reducing the total NO-dependent vasorelaxation or due to compensatory increased NO-independent vasodilator mechanisms [27]. On the other hand, the inhibition of sGC significantly blocked the EDR in all age groups, indicating important sGC involvement in these vasoactive responses. Recent studies implicated nitroxyl (HNO) as another possible activator of sGC whose action is independent of NO formation [28, 29]. Andrews et al. [30] demonstrated that HNO is produced endogenously and serves as an endothelium-derived relaxing and hyperpolarizing factor in both mouse and rat isolated small mesenteric resistance-like arteries. However, information about the age-related changes in HNO production and its vasoactive properties is limited. According to this, we assume that aging in the WKY could probably be associated with enhanced NO production (increased NOS activity in the old WKY) and bioavailability (comparable superoxide concentrations among age groups); indeed, the smallest L-NMMA-sensitive component of EDR was confirmed in aged WKY; in order to prevent the additional deterioration of EDR and SBP increase.

Interestingly, in the young SHRs, the EDR was increased compared to the age-matched WKYs, while in the other age groups, a significantly reduced EDR was observed. Since endothelial dysfunction resulted in a shift of endothelium-derived vasoactive agents toward vasoconstrictors, our previous studies were aimed at testing the effect of cyclooxygenase (COX) inhibition on EDR in SHRs. We confirmed that specific COX-2 inhibition recovered the reduced EDR in 20-week-old and 52-week-old SHRs [31, 32]. Thus, COX-2-derived vasoconstrictor prostaglandins are significantly involved in the development of endothelial dysfunction in SHRs. Apart from this, a reduced bioavailability of NO has often been reported as a main factor of impaired EDR in SHRs. Our present results showed that in 7-week-old SHRs, the residual EDR after L-NMMA incubation was increased compared to that of rats in other age groups, but rats at 20 weeks and 52 weeks had similar residual EDRs. The activity of NOS in the TA was comparable in 7-week-old and 20-week-old SHRs, but it was significantly reduced in 52-week-old hypertensive rats compared to younger animals and 52-week-old WKYs. According to these data, it seems that in SHRs, the lowest aortic NO bioavailability was in the 52-week-old group.

A comparison of these responses between the strains revealed that the 7-week-old SHRs had significantly enhanced residual relaxation after L-NMMA incubation compared with the age-matched WKYs, indicating a decreased L-NMMA-sensitive component of the EDR in SHRs. On the other hand, NOS activity and superoxide production were significantly increased in the 7-week-old SHRs compared to age-matched WKY rats. In our previous study, we confirmed that maximal acetylcholine-induced vasorelaxation was inhibited by a nonspecific NOS inhibitor (L-NAME) to a higher extent in 4-week-old SHRs than in age-matched normotensive rats. Moreover, a higher concentration of L-NAME induced a significant increase in TA basal tone in SHRs, which was comparable with submaximal noradrenaline-induced vasoconstriction [10]. These findings indicate the prevalent participation of NO in vasoactive control of prehypertensive and early hypertensive stages of SHRs and support the existence of adaptation mechanisms involving abundant endogenous vascular NO production [12]. This was also observed previously in the femoral arteries, in which endothelial dysfunction in SHRs was NO-independent, while there was a significant negative correlation between BP and the L-NAME-resistant component of ACh-induced relaxation in young adult rats with various genetic predispositions to hypertension [33]. These adaptation mechanisms are functional in 7-week-old SHRs, since we also found enhanced NOS activity in the aorta compared to 7-week-old male WKYs in this study and previously in age-matched female SHRs [16]. In the present study, the increased NOS activity persisted in 20-week-old SHRs; however, the EDR was reduced, and the residual EDR after L-NMMA was the same as that in age-matched WKYs. Therefore, it seems that in young adult SHR there is a sufficient amount of vasoactively relevant NO in TA. However, in aged SHRs, the activity of the L-arginine/NO pathway in TA decreased, which was not observed in WKY rats. Thus, it seems that in contrast to SHRs, in WKYs, the NOS/NO system is probably able to respond to age-related pathologies to maintain endothelial functions and thus optimal BP levels even in later periods of life.

## 5. Conclusions

In summary, our results demonstrated that sGC is the dominant target of endogenous NO in both normotensive and hypertensive conditions and that age did not alter its importance in the EDR of TA. However, the production and participation of vascular NO in EDR is clearly affected not only by hypertension but also by age. While young juvenile SHRs revealed enhanced NOS activity and vasomotor manifestations of NO compared with age-matched WKYs, these benefits partially disappeared in young adult rats (enhanced NOS activity but EDR reduction). Moreover, in old SHRs, there was an obvious reduction in the activity of the L-arginine/NO pathway in the TA, which was not found in the WKY rats. We can conclude that in WKYs, the NOS/NO system is probably able to respond to age-related pathologies in an effort to maintain endothelial functions and normotension, which is missing in aged hypertensive rats.

## Figures and Tables

**Table 1:** General biometric and cardiovascular parameters.

	WKY-7	SHR-7	WKY-20	SHR-20	WKY-52	SHR-52
BW (g)	144 ± 4	145 ± 5	359 ± 5^+^	350 ± 6^+^	447 ± 8^+x^	426 ± 3^+x^^∗^
SBP (mmHg)	117 ± 1	160 ± 3^∗^	119 ± 3	204 ± 6^+^^∗^	126 ± 1^+x^	198 ± 3^+^^∗^
HR (bpm)	479 ± 14	547 ± 9^∗^	463 ± 16	561 ± 13^∗^	483 ± 12	559 ± 17^∗^
LV (mg)	265 ± 11	308 ± 15	596 ± 16^+^	745 ± 25^+^^∗^	691 ± 16^+x^	883 ± 19^+x^^∗^
TL (mm)	25.88 ± 0.24	25.81 ± 0.23	37.06 ± 0.16^+^	36.46 ± 0.14^+^^∗^	39.44 ± 0.20^+x^	39.06 ± 0.09^+x^
LV/TL (mg/mm)	10.21 ± 0.39	11.91 ± 0.50^∗^	16.09 ± 0.45^+^	20.42 ± 0.62^+^^∗^	17.52 ± 0.38^+x^	22.60 ± 0.50^+x^^∗^
LV/BW (mg/g)	1.84 ± 0.08	2.08 ± 0.08^∗^	1.66 ± 0.05	2.13 ± 0.07^∗^	1.55 ± 0.05^+^	2.07 ± 0.05^∗^

BW: body weight; SBP: systolic blood pressure; HR: heart rate; LV: weight of the left heart ventricle; TL: tibia length; LV/TL: ratio of weight of the left heart ventricle to tibia length; LV/BW: ratio of weight of the left heart ventricle to body weight; WKY-7, WKY-20, and WKY-52: 7-week-old, 20-week-old, and 52-week-old Wistar-Kyoto rats; SHR-7, SHR-20, and SHR-52: 7-week-old, 20-week-old, and 52-week-old spontaneously hypertensive rats. The results are presented as the mean ± SEM of *n* = 9–14 rats, and differences between groups were analyzed by two-way analysis of variance (ANOVA) with the Bonferroni post hoc test on ranks. ^∗^*p* < 0.05 vs. WKY at the same age (WKY-7 vs. SHR-7, WKY-20 vs. SHR-20, and WKY-52 vs. SHR-52), ^+^*p* < 0.05 vs. 7-week group of the same phenotype (WKY-20 vs. WKY-7, SHR-20 vs. SHR-7, WKY-52 vs. WKY-7, and SHR-52 vs. SHR-7), ^x^*p* < 0.05 vs. 20-week group of the same phenotype (WKY-52 vs. WKY-20 and SHR-52 vs. SHR-20).

## Data Availability

The data used to support the findings of this study are available from the corresponding author upon request.
